# Mr. Smith on Scarlatina

**Published:** 1812-02

**Authors:** Henry Smith

**Affiliations:** Salisbury


*To the Editors of the Medical and Physical Journal.*

GENTLEMEN,

GENTLEMEN,

THE Scarlatina having prevailed for these three months past, and continuing to prevail, in this city and its neighbourhood, I am induced to offer a few observations upon it, and the methodus medendi, by bleeding, of Mr. Machell, in your last month's Journal; at the same time I will endeavour to establish a criterion to guide the inexperienced in what cases bleeding may be admissible, and when, in all probability, hurtful. If you should consider my observations worth insertion in your useful Journal, they are at the service of the public. I beg to assure you, that I make them with much deference and hastily, under the pressure of various avocations. Perhaps some future opportunity I may detail some cases and the treatment pursued; at present I am concerned to establish a distinction, in the species of Scarlatina, that may lead to utility, and more certainty in practice, and, if possible, reconcile the discrepances of authors on this disease. I am, Gentlemen,

Your obedient Servant,

*Salisbury, Dec.* 11, 1811. HENRY SMITH.

Having

Having the medical care of the poor of this city and three circumjacent parishes, together with extensive private practice, I have, consequently, many and frequent opportunities of seeing patients with Scarlatina, in all its varieties, under all circumstances of poverty and the means of procuring comforts, in all constitutions both of children and adults. I may therefore be allowed to speak experimentally, and not to incur the censure passed by the venerable and sagacious Cullen on case-framers. * Plures denique ut observationibus, hodie adeo expetitis, famam sibi compararent, historias in museo fictas pro veris tradiderunt.

I have lived long enough to see the rise and fall of theories and of medicines; among many others may be noticed the specious system of Brown, and the fanciful hypothesis of Darwin: yet I cannot forbear to pay a tribute to the memory of the latter, that, however fanciful may be his theories, we must allow his practical rules are excellent.

I wish to speak with much deference of the practice adopted by others, yet I must be allowed to consider the indiscriminate use of the lancet in Scarlatina, without pointing out its varieties, as calculated to mislead and do much mischief. I shall take the liberty to divide Scarlatina into two species: the first I would name Scarlatina Tonsillitis †, and will proceed to describe what I mean under this title, particularly as it appeared among my patients. It began with the usual symptoms of fever, viz. rigors, head-ache, pains of the limbs, particularly the loins; generally, not always, sickness of the stomach, from which bile was vomited, constipation, oftentimes diarrhœa, thirst; about the fourth day some tumefaction of the face, redness of the eyes, delirium, and a vivid efflorescence on the skin of the whole body, more or less; oppression about the præcordia, with tenderness on pressing it, particularly before the efflorescence, and generally going off when that made its appearance; heat of the body very much increased. On inspecting the fauces, the tonsils, uvula, and velum pendulum palati, together with the lining membrane of the mouth and tongue, were found swelled and inflamed, covered more or less with white crusts (not aphthæ ‡) raised above the surface, and which I consider to be the coagulable lymph denoting membranous inflammation; and, when they fall off, leaving no ulceration or

* Nosol. Method. Prolog. P. vi.

† I am aware Tonsillitis is not a legitimate term, but I know of no other so appropriate.

‡ Aphthæ I would describe as ulcuscles, not raised above the surface, covered with a white film, concealing ulceration, and always denote debility with the typhoid state of the system.

loss loss of substance, they are frequently spit up, and are described by the patient like bits of flesh.*

The tongue, from being white, frequently appears vividly red, as described by Dr. Willan, on cutaneous diseases. The tonsils were sometimes so enlarged as nearly to fill the cavity of the throat, and then the patient complained of great anxiety, restlessness, and oppression ; sometimes matter formed in the tonsils, which, breaking, a considerable alleviation of all the symptoms followed. If it were not for the efflorescence of the skin, the more diffused and greater number of white crusts, and the more pressing symptoms of fever, it would resemble the Cynanche tonsillaris. About the third, fourth, or fifth, day, seldom later in the inflammatory species, desquamation of furfuraceous scales takes place, accompanied with a distressing pruritus, leaving the patient very weak, sometimes anasarcous with dyspnœa †, and a troublesome cough. This species of Scarlatina appears to me to be attended with the Synochus of Cullen.

The second species I would distinguish by the name of Scarlatina Maligna, and is attended by a considerable increase of all the symptoms of the former, particularly delirium, restlessness, and anxiety, but differing in the appearance of the tonsils, fauces, and tongue. The tonsils are not near so much swelled, yet the difficulty of swallowing and tenderness are equally great. They appear of a much darker red, and are ulcerated. The patient's breath is very fetid, and the stools highly offensive. Aphthous ‡ ulcuscles are diffused around and over the mouth, the tongue is very foul, the functions of the brain are much disturbed, vomiting and diarrhœa, attended with great prostration of strength. The efflorescence on the skin is of a darker hue than in the former, nor does desquamation take place so soon. I have frequently observed the rash disappear without any desquamation. From about the seventh to the twelfth day anasarca comes on, frequently with considerable pain and inflammation in the legs, accompanied either with petechiæ or a troublesome itching eruption. In some cases there was an eruption about the lips and inside of the nose. This species is attended with the Typhus of Cullen. I have seen both species assume all the intermediate shades from Synochus to Typhus mitior et gravior.

It manifestly appears to me, that the two scarlatinas here described, required very different treatment. In the first

* The crustæ albidæ may generally be observed in the Cynanche Tonsillaris, and accompany almost all inflammations of membranes.

† From Anasarca of the lungs.

‡ See former description of aphthæ.

we may bleed and use evacuants with the prospect of success, and adopt what is called the antiphlogistic regimen; in the latter the same treatment would, I think, be destructive.

Among the number of patients that have fallen to my lot in this fever I have not lost one, yet I have not practised bleeding upon any. In the Scarlatina Tonsillitis I have used vomits and purges, blisters, and particularly the cold affusion with refrigerants. In the Scarlatina Maligna I have pursued a different plan, mutatis mutandis, according to the shades of difference. In cases where I was called in early, I generally began with a vomit; and, when the diarrhœa was considerable, I had immediately recourse to cordials and astringents, with opiates, and a moderate use of wine *, according to the debility, taking care not to administer it so as to increase the delirium or produce intoxication. If the diarrhœa was not great, a few grains of rhubarb generally succeeded the vomit, and then the cordial plan with the free admission of fresh air, nitrous fumigation, and lavation with *warm* vinegar and water. I will just mention, by way of illustration, the cases of three patients, in a poor family, at Laverstock, near this city. The first, a stout girl about 16 years old, of the sanguine temperament, had been ill three or four days before I saw her, with Scarlatina Tonsillitis. Her brother, a lad of the phlegmatic temperament, about 14 years old, was seized about two days after her with the Scarlatina Maligna. They both recovered, under very different treatment. Another brother, a stout lad about 18, resembling the sister, complained whilst I was attending the former: I immediately vomited and purged him, and by these means arrested the progress of disease, as he was well again in three or four days. I am at this time attending a poor man, a weaver, aged 35, of the phlegmatic temperament, who has a family of seven children; two of them have recovered from Scarlatina Tonsillitis about a fortnight since, the rest have hitherto escaped, although there is but one sleeping-room for the whole party. Small doses of calomel and antimonial powder, sufficient to keep up a gentle diarrhœa, were the only medicines required for them. The father, about a week ago, was seized with all the symptoms of Scarlatina Maligna, except the efflorescence on the skin: the tonsils were ulcerated, and but little swelled; tongue very foul; delirium very slight, but considerable anxiety and restlessness, with as much difficulty in swallowing

* The bark was seldom had recourse to early, not indeed till there was some abatement of delirium and remission of fever. The sulphuric acid conjoined with opium and camphor answered better.

as if the tonsils filled the cavity of the throat; his bowels at first were constipated, and a small dose of rhubarb, after a vomit, producing only three stools, occasioned him to faint; his pulse is under 100 beats in a minute, and the general appearance of the symptoms is that of Typhus mitior. The præcordia is very tender on pressure *, and I think we must allow this to be a modification of Scarlatina Maligna, produced by the same kind of contagion that affected his two children, but modified by constitution. My case-book would furnish me with many other examples illustrative of these observations, but the above may suffice to shew that the same contagion will produce different effects upon different temperaments of constitution, and consequently requiring a very different treatment of the disease.

Before I examine Mr. Machell's cases, I will proceed to make some observations on the difference of practice in different places, and the discrepances of constitution in high and low lands. This city is situated in a valley, I may say a meadow, surrounded with water, which is conducted by channels through the streets: it is also a manufacturing city, and most of the poor are weavers, confined to dirty and close rooms, leading sedentary lives, and are great tea-drinkers. I have observed, for some years past, that the inhabitants generally do not bear bleeding, even in decided inflammatory cases, to the extent recommended by some authors. Whether this be the case in similar low situations, I cannot tell; but to account for it I have conjectured, that the purity of the air is diluted by marshy exhalations, as well as the unwholesome occupations of the inhabitants, and that the oxygen is consequently less stimulating. The subjects of Mr. M.'s cases were situated high above the level of water, and consequently free from marshy exhalations to dilute the purity of the air; the oxygen of course being more pure, the inflammatory diathesis would prevail in the constitutions of the inhabitants, and they would require and bear bleeding much better than in low and less favourable situations. It would be particularly useful, and contribute very much to the improvement of medicine, if some person or persons, properly qualified, would give us a medical and topographical survey of the different counties in England. At present it is difficult, but upon some such supposition as before stated, to reconcile the wide difference in the treatment of diseases and epidemics, and effects of remedies. Whoever is conversant with the practice of the ancients, both of other countries and our own, as well as the moderns of continental

* I have often observed this before the efflorescence appeared.

Europe

Europe and other parts of the world, cannot fail to observe the discrepances of the *methodus medendi* of different authors, particularly with regard to epidemics. Some recommend bleedings even to an extent that would startle Sangrado himself, and with as little discrimination; others insist upon bark, wine, and cordials, "stimulating their patients to death." As far as my observations go, I scarcely find two patients that can be treated exactly alike under the same nomenclature of disease. In answer to Mr. M. respecting the virulence of the Scarlatina in his neighbourhood, I believe we may not wander far to account for it. It appears evidently to owe its virulency to the improper management of the sick before he was called in. He states, that the patients were kept to their beds, in confined rooms, without the necessary renovation of fresh air and ventilation, and in the midst of summer. I conceive this would be sufficient to render any epidemic most malignant, in any situation of place or persons.

It will be needless, and lengthen this article beyond bounds, to multiply quotations in support of these observations, I will therefore be content to refer your readers to Sydenham and Huxham, to Van Swieten in Boerhaavii Aphorismos, to Fothergill and Cullen, particularly the notes of the latter, in his Nosol. Method., and to the learned and ingenious Dr. Willan on Cutaneous Diseases, wherein every thing relating to Scarlatina is set forth; the bleeding system of Rush, and the stimulating of others. Whoever is desirous of further investigating the history of bleeding in different diseases, may consult the elaborate treatise of Horatius Augenius de missione sanguinis, wherein are discussed the practice and opinions of Hippocrates, Galen, &c. &c.

I will now proceed to Mr. M.'s cases, and I beg him to consider my comments thereon as made with much deference to him; and the only object I have in view, is to join him and others in rendering the history and treatment of diseases, particularly epidemics, more accurate. Should he again favor us with cases, he will, I hope, more clearly distinguish the species of Scarlatina, and at the same time define what he means by sore throat and aphthæ.

Case 1.—In this case Mr. M. appears to have acted very judiciously, particularly with respect to the admission of fresh air, ventilation, changing bed and bed-clothes, &c. &c. after the "room had been previously kept very close." I am disposed to consider the abatement of fever and delirium, and ultimate recovery of the patient, to the judicious alteration in his apartment, and the sponging with cold water,

 rather rather than to the abstraction of twelve ounces of blood. Indeed, I think it appears clearly, from the narrative, that the cold affusion and admission of cool air produced the favorable result, for the V. S. was not repeated, although the delirium and other symptoms, after the effect of the first ablution had subsided, increased. This may be considered as an example of the Scarlatina Tonsillitis. The mention of "sore throat" is very indefinite, for it may arise as well from pure inflammation of the tonsils as ulceration.

The aphthæ were probably the crustæ albidæ before described.

Case 2.—Similar to the last, but much slighter, owing, perhaps, to the early application of proper remedies.

Case 3.—Scarlatina Maligna.

Case 4.—An adult, of a "strong constitution and full habit," that is, of the sanguine temperament, with "inflamed tonsils," but no appearance of what he calls aphthæ or ulceration—"face humid, eyes very red."—"Sore throat" is here again an indefinite term. In this case bleeding and calomel performed the cure, as may reasonably have been expected in such a habit.

Case 5.—I suppose a similar constitution to the former. His body covered with "bright red" efflorescence; "face swollen; throat, tongue, and palate, covered with *aphthæ;* pulse full and rapid," eyes very red; all clearly symptoms of inflammatory action; no mention of ulcerated tonsils. This I should consider to be another example of the Scarlatina Tonsillitis, in which the treatment was judicious, and, as might be supposed, curative. The other cases appear to be examples of the Scarlatina Tonsillitis: many such have occurred among my patients. I have never found bleeding necessary; evacuants and refrigerants have succeeded to my wish; and, as I have before stated, not a patient has died hitherto, during the reign of Scarlatina in this place. It may be observed that I have not mentioned the pulse in the enumeration of symptoms; the reason is, it has varied so much, that no correct judgment could be formed from it, and I have been guided in general by the other symptoms. My experience will allow me to bear testimony to the truth of Mr. Burns' observation, that, "in acute diseases, it is a very bad sign to find the pulse full and the beat very frequent; for this marks that the artery is unable fully to contract."—*Dissertations on Inflammation,* vol. i. p. 94.

